# Socioeconomic disparities in the association of age at first live birth with incident stroke among Chinese parous women: A prospective cohort study

**DOI:** 10.7189/jogh.14.04091

**Published:** 2024-04-08

**Authors:** Weidi Sun, Shiyi Shan, Leying Hou, Shuting Li, Jin Cao, Jing Wu, Qian Yi, Zeyu Luo, Peige Song

**Affiliations:** School of Public Health and the Second Affiliated Hospital, Zhejiang University School of Medicine, Hangzhou, Zhejiang, China

## Abstract

**Background:**

Stroke has become a significant public health issue in China. Although studies have shown that women’s age at first live birth (AFLB) might be associated with incident stroke, there is limited evidence on this relationship among Chinese parous women. Likewise, the nature of this association across urban-rural socioeconomic status (SES) has yet to be explored. In this prospective study, we sought to investigate the associations of women’s AFLB with the risk of incident stroke and its subtypes (ischaemic stroke, intracerebral haemorrhage, and subarachnoid haemorrhage) and to explore the differences of these associations as well as the population-level impacts across SES classes.

**Methods:**

We used data on 290 932 Chinese parous women from the China Kadoorie Biobank who were recruited in the baseline survey between 2004 and 2008 and followed up until 2015. We used latent class analysis to identify urban-rural SES classes and Cox proportional hazard regression to estimate hazard ratios (HRs) and 95% confidence intervals (CIs) for AFLB’s association with incident stroke. We then calculated population attributable fraction (PAF) to demonstrate the population-level impact of later AFLB on stroke.

**Results:**

Around 8.9% of parous women developed stroke after AFLB. Compared with women with AFLB <22 years, those with older AFLB had a higher risk of total stroke, with fully adjusted HRs (95% CI) of 1.71 (95% CI = 1.65–1.77) for 22–24 years and 3.37 (95% CI = 3.24–3.51) for ≥25 years. The associations of AFLB with ischaemic stroke were stronger among rural-low-SES participants. We found the highest PAFs of ischaemic stroke (60.1%; 95% CI = 46.2–70.3) associated with later AFLB for urban-high-SES individuals.

**Conclusions:**

Older AFLB was associated with higher risks of incident stroke and its subtypes among Chinese parous women, with stronger associations between AFLB and ischaemic stroke among rural-low-SES participants. Targeted medical advice for pregnant women of different ages could have long-term benefits for stroke prevention.

Stroke has emerged as a major public health concern, ranking as the second-leading cause of death globally. In 2019, it was responsible for 11.6% of total mortality, translating to 6.55 million deaths [1,2]. In China specifically, the number of deaths due to stroke has increased by 59.0% over the past decades, reaching 2.19 million in 2019 [2,3]. This alarming trend highlights the need for targeted preventive measures. Stroke manifests mainly as ischaemic or haemorrhagic, with each subtype having distinct pathophysiological profiles and risk factors [2]. Ischaemic stroke occurs due to blockages in the arterial system, often from thrombosis or embolism, while haemorrhagic stroke results from the rupture of blood vessels, commonly influenced by hypertension and aneurysms [4]. Due to the distinct pathophysiological mechanisms of stroke subtypes, there is a need to better understand the diverse risk factors of stroke subtypes.

Pregnancy and delivery represent significant events in women’s lives, during which biological and physiological changes like cardiac hypertrophy, increased insulin resistance, abnormal lipids, and elevated inflammation could substantially impact maternal health [5–8]. The age at first live birth (AFLB) is a critical reproductive factor impacting women’s health [9]. From the biodevelopmental perspective, early AFLB soon after puberty is recommended to capitalise on the peak functional capacity of the reproductive system [10]. In fact, later AFLB may lead to increased maternal and perinatal risks, such as spontaneous abortion, gestational diabetes, hypertensive disorders, and preeclampsia [11], which can affect immediate pregnancy and delivery outcomes, and also result in long-term impacts on maternal health, potentially increasing the risk of cardiovascular diseases (CVDs) [12].

Studies have reported mixed findings on the associations between AFLB and subsequent CVDs, including stroke. For instance, the significant association between later AFLB and a reduced risk of CVDs observed in the Nurses’ Health Study II disappeared after adjusting for sociodemographic and health-related covariates [11]. Meanwhile, the UK Biobank study found an inverse association between AFLB and stroke risk, which diminished after adjusting for socioeconomic status (SES) and lifestyle factors [13]. However, there are still few studies, especially longitudinal ones, exploring this association in Chinese women.

As shown previously, the association between AFLB and stroke risk could be further complicated by SES, a multifaceted indicator of an individual’s social position [14,15]. Specifically, early AFLB may lead to reduced employment opportunities and disruptions in educational or career trajectories, resulting in poorer health outcomes [16]. Conversely, women with later AFLB might benefit from healthier lifestyles and a stronger sense of personal control, which might decrease their CVD risks [17]. With the currently rising trend of delayed childbirth, especially among women with higher SES, the interaction between reproductive decisions and later-life health outcomes has become increasingly relevant [16,18]. Additionally, considering the prevalent urban-rural disparities in China, there may exist variations in AFLB and the susceptibility to stroke between urban and rural residents [19,20]. Therefore, exploring the moderating role of urban-rural SES in the association between women’s AFLB and the risk of stroke could prove worthwhile.

In this study, we used data from the China Kadoorie Biobank (CKB) to investigate the associations between AFLB and the incidence of ischaemic and haemorrhagic strokes. We wanted to explore socioeconomic disparities in these associations and how different SES levels might influence the stroke burden related to AFLB. We hypothesised that there is a significant link between AFLB and stroke burden, which may be more pronounced among women of lower SES and in rural settings. By addressing these research gaps, we strive to inform targeted prevention strategies and contribute to improved health outcomes for women across diverse socioeconomic landscapes in China.

## METHODS

### Study population

The CKB study is a nationwide, prospective cohort study of the Chinese population; its design and methodology have been reported elsewhere [21,22]. Briefly, 512 726 adults aged 30–79 years were recruited from 10 (five urban and five rural) geographically diverse regions across China at the baseline survey between June 2004 and July 2008, with subsequent follow-up surveys conducted at intervals of approximately 4–5 years. Trained full-time staff collected information through laptop-based questionnaires, physical measurements, and blood tests. The CKB study obtained ethical approval from the Oxford University Tropical Research Ethics Committee (Approval No. 025-04) and the Chinese Center for Disease Control and Prevention Ethical Review Committee (Approval No. 005/ 2004). All participants provided written informed consent.

**Figure 1:**
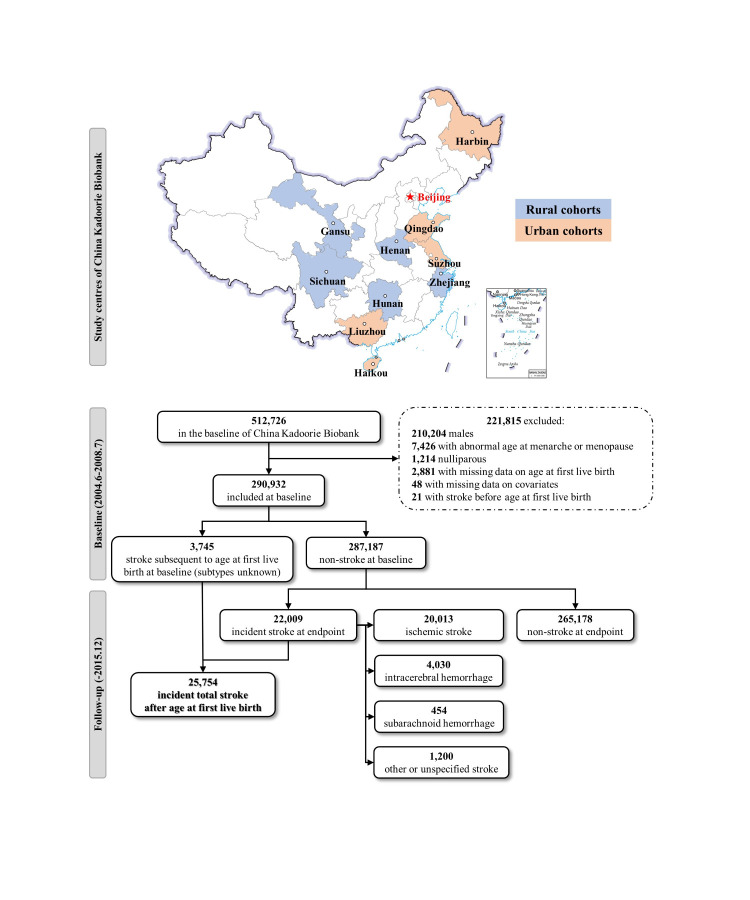
Flowchart of participants' inclusion. Time-to-event variable was calculated from age at first live birth to age at stroke occurrence. Total strokes included incident strokes after age at first live birth at baseline and during follow-up, whichever came first. Subtypes of strokes at baseline were ambiguous.

To obtain our sample, we excluded participants who were males (n = 210 204); had abnormal age at menarche (<9 or >20 years) or menopause (<40 years or more than the individual’s actual age at baseline) (n = 7426); nulliparous (n = 1214); had missing data on AFLB (n = 2881) or covariates (n = 48); or had a stroke prior to first parturition (n = 21), leaving 290 932 women for further analysis (**Figure 1**). This meant that the sample fulfilled the requirement of a minimum of 6536 participants, as determined by the sample size calculation formula for longitudinal studies [23].

### Assessment of age at first live birth

Reproductive histories, including live birth counts and age at each live birth, were collected at the baseline of CKB through an interviewer-administered electronic questionnaire. AFLB was defined as the age of a parous woman at which she gave birth to the first live child, which was accurate to one year. We categorised AFLB into <22 years, 22–24 years, and ≥25 years, according to tertiles.

### Ascertainment of incident strokes

The primary outcome was an incident stroke which occurred after AFLB. At the baseline of the CKB survey, participants self-reported their physician’s diagnosis of stroke history (whose subtypes were ambiguous) and age at the time of the stroke event. During follow-ups, information on incident stroke was collected through linkage with the disease and mortality surveillance system and the national health insurance claim database, with almost universal coverage across the ten study areas (>98%). Strokes during follow-up were coded according to the International Classification of Diseases, 10th Revision (I63 for ischaemic stroke, I61 for intracerebral haemorrhage, I60 for subarachnoid haemorrhage, and I64 for other unspecified stroke). Total strokes included incident strokes after AFLB at baseline and during follow-up, whichever came first.

### Measurement of covariates

Based on previous literature, we involved a range of covariates that would potentially impact the association between AFLB and stroke: Age, residence, lifestyle behaviours (smoking, drinking, and physical activity), passive smoking history, reproductive factors (marital status, parity, and menopausal status), medical history (hypertension, diabetes, and coronary heart disease), medication history (oral contraceptive pills usage, anticoagulation therapy, and hypolipidemic therapy), socioeconomic variables (annual household income, education, and occupation), and anthropometric data (height, weight, and waist circumference (WC)) [9]. Information on confounders other than anthropometric indicators was collected through questionnaires at baseline during 2004–08. Residence was divided into rural and urban. Smoking was categorised as ‘never smoker,’ ‘ever smoker,’ and ‘current smoker,’ and drinking as ‘never drinker,’ ‘ever drinker,’ and ‘current drinker.’ Physical activity was measured by multiplying metabolic equivalent of task (MET) values by hours spent on all daily activities. Passive smoking history, oral contraceptive pills usage, anticoagulation therapy, and hypolipidemic therapy were dichotomised into ‘yes’ and ‘no.’ Likewise, marital status was dichotomised to ‘yes’ (married) and ‘no’ (not married conditions, including never married, divorced, separated, or widowed), as was menopausal status (‘yes’ if currently having or had menopause, ‘no’ otherwise). Parity, which referred to the number of live births, was further categorised as 1, 2, 3, and ≥4. Annual household income in CNY was categorised as <10 000, 10 000–34 999, and ≥35 000. Education was categorised as ‘primary school and below,’ ‘middle school,’ and ‘high school and above.’ Occupation was grouped as ‘unemployed, retired, or others,’ ‘farmer or worker,’ and ‘sales, self-employed, manager, or professional.’

Physical examinations were simultaneously conducted by uniformly trained technicians to obtain height, weight, WC, blood pressure, and blood glucose. Height and weight were measured to the nearest 0.1 cm and 0.1 kg, respectively. Body mass index (BMI) was calculated by dividing weight (kg) by the square of height (m^2^) and was divided into four categories: underweight (<18.5 kg/m^2^), normal (18.5–23.9 kg/m^2^), overweight (24.0–27.9 kg/m^2^), and obesity (≥28 kg/m^2^) [24]. WC was recorded with an accuracy of 0.1 cm and treated as a continuous variable. Blood pressure was measured twice, and the average was recorded. If the difference in systolic blood pressure was greater than 10 mm Hg, a third measurement would be conducted, and the average of the last two measurements would be recorded. Hypertension was defined as a blood pressure ≥140/90 mm Hg, or a self-reported physician diagnosis or under treatment. Random blood glucose was assessed by a glucometer (Johnson SureStep Plus). Diabetes was defined as fasting glucose ≥7.0 mmol/L, random glucose ≥11.1 mmol/L, or a self-reported physician diagnosis or receiving treatment.

### Statistical analysis

As all continuous variables in the analyses were highly skewed in skewness-kurtosis normality test, we reported them as median and interquartile ranges (IQRs). We otherwise presented categorical variables as numbers and percentages, and described baseline characteristics by stroke status. We calculated the incidence rate of total stroke, ischaemic stroke, intracerebral haemorrhage, and subarachnoid haemorrhage by AFLB categories as the number of events per 1 000 000 person-years.

We conducted Cox proportional hazard regression stratified by birth years to estimate hazard ratios (HRs) and 95% confidence intervals (CIs) of AFLB categories for incident stroke and its subtypes, evaluating the proportional hazards assumption by Schoenfeld’s test. We calculated the time-to-event variable from AFLB to the age at the first incident stroke, loss to follow-up, or 31 December 2015 (the endpoint). We then established four multivariable models: 

1. Model 1 was adjusted for age at baseline;

2. Model 2 was further adjusted for BMI categories, WC, smoking, passive smoking, drinking, physical activity, marital status, the history of diabetes, hypertension, coronary heart disease, oral contraceptive pills usage, history of anticoagulation therapy and hypolipidemic therapy, menopausal status, and parity based on model 1;

3. Model 3 was further adjusted for residence based on model 2;

4. Model 4 was further adjusted for education, occupation, and annual household income based on model 3.

To discover the SES disparities in the association, we used latent class analysis (LCA) method to explore underlying SES classes within observed data based on three SES variables (including annual household income, education, and occupation) [25]. Specifically, we conducted separate analyses for urban and rural areas. As a person-centred methodological approach, LCA aims to separate distinct subgroups within the entire participants based on the population heterogeneity and helps to assign subgroup labels inferred from patterns of observed variables. It is a form of mixture modelling considered a more statistically robust method of clustering and has been widely used in identifying different phenotypes and socioeconomic patterns [26,27]. Here we selected the optimal fit of the classing model based on the theoretical interpretability and fit statistics, including the low absolute Akaike information criterion (AIC) and Bayesian information criterion (BIC) values, as well as high entropy (Table S1 in the **Online Supplementary Document**) [28]. Considering the substantial urban-rural disparities in China [19,20], we categorised the socioeconomic classes by both individual SES and urban-rural residence. Finally, we extracted two latent SES classes in urban areas and identified them as high and low SES; in rural areas, we extracted three latent SES classes representing high, medium, and low SES. We determined class membership by the posterior item probabilities. Characteristics of participants across the urban-rural SES classes were compared through the Wilcoxon rank sum test for continuous variables and χ^2^ test for categorical variables. We used Kaplan-Meier (KM) curves to visualise the survival probability free from stroke among individuals with different AFLB, stratified by SES classes.

We then conducted SES-stratified analysis with adjustment of covariates in the model 2 mentioned above. We examined the interaction of urban-rural SES classes with AFLB by comparing models with and without a multiplicative interaction term of SES classes and AFLB categories to obtain *P*-values for interaction. To verify the robustness of the results, we performed subgroup analyses among rural and urban residents, separately. We estimated the rural-to-urban ratio of HR to compare the effect differences between rural and urban areas [29] and investigated the associations within specific categories of each SES component (i.e. annual household income, education, and occupation).

Finally, we calculated the population attributable fraction (PAF) to demonstrate the population-level impact of later AFLB on stroke based on the unrealistic counterfactual scenario [30]. For this purpose, we used the ‘punaffc’ package in Stata, which is suitable for survival data [31].

We performed all analyses in Stata, version 16.0 (StataCorp LLC, College Station, TX, USA). A two-sided *P*-value <0.05 or a 95% CI that did not cross 1.00 denoted statistical significance.

## RESULTS

### Participants’ characteristics at baseline

Of the 290 932 included parous women, 3745 developed stroke at baseline subsequent to AFLB; 22 009 developed stroke during the follow-up (20 013 ischaemic strokes, 4030 intracerebral haemorrhages, and 454 subarachnoid haemorrhages), with a median survival time of 34.7 years. The median ages at baseline of parous women developing incident ischaemic stroke, intracerebral haemorrhage, and subarachnoid haemorrhage were 60.0 (IQR = 52.9–67.3) years, 60.1 (IQR = 52.7–67.9), and 57.0 (IQR = 50.3–64.8), and were thus notably higher than the age of women without stroke (50.0 years, IQR = 42.1–57.4). Regarding AFLB, 35.4% of parous women with stroke had their first live delivery <22 years, 31.4% between 22 and 24 years, and 33.2% at 25 years and above. Meanwhile, 28.6% of parous women without stroke had their first delivery at <22 years, 38.3% at 22–24 years, and 33.0% at and ≥25 years (**Table 1**). The incidence rates of stroke by AFLB groups are shown in Table S2 in the **Online Supplementary Document**.

**Table 1:** Baseline characteristics of included parous women

		Women with incident stroke			
Baseline characteristics*	**Women without incident stroke (n = 265 178)**	**Total stroke (n = 25 754)**†	**Ischaemic stroke (n = 20 013)**	**Intracerebral haemorrhage (n = 4030)**	**Subarachnoid haemorrhage (n = 454)**
**Age in years, median (IQR)**	50.0 (42.1–57.4)	60.0 (52.9–67.4)	60.0 (52.9–67.3)	60.1 (52.7–67.9)	57.0 (50.3–64.8)
**Residence**					
Rural	149 929 (56.5)	12 042 (46.8)	8583 (42.9)	2973 (73.8)	260 (57.3)
Urban	115 249 (43.5)	13 712 (53.2)	11 430 (57.1)	1057 (26.2)	194 (42.7)
**Education**					
Primary school and below	148 420 (56.0)	16 267 (63.2)	12 220 (61.1)	3156 (78.3)	296 (65.2)
Middle school	69 118 (26.1)	5236 (20.3)	4203 (21.0)	549 (13.6)	101 (22.2)
High school and above	47 640 (18.0)	4251 (16.5)	3590 (17.9)	325 (8.1)	57 (12.6)
**Occupation**					
Unemployed, retired and other	92 797 (35.0)	16 075 (62.4)	12 660 (63.3)	2075 (51.5)	227 (50.0)
Farmer or worker	141 585 (53.4)	8265 (32.1)	6132 (30.6)	1838 (45.6)	202 (44.5)
Sales, self-employed, manager or professional	30 796 (11.6)	1414 (5.5)	1221 (6.1)	117 (2.9)	25 (5.5)
**Annual household income in CNY**					
<10 000	77 340 (29.2)	8497 (33.0)	6242 (31.2)	1841 (45.7)	138 (30.4)
10 000–34 999	143 046 (53.9)	13 819 (53.7)	10 939 (54.7)	1850 (45.9)	260 (57.3)
≥35 000	44 792 (16.9)	3438 (13.3)	2832 (14.2)	339 (8.4)	56 (12.3)
**BMI in kg/m^2^, median (IQR)**	23.5 (21.4, 25.8)	24.5 (22.1, 27.0)	24.6 (22.2, 27.1)	23.7 (21.2, 26.5)	23.7 (21.5, 26.2)
**BMI categories**					
Underweight	11 268 (4.2)	947 (3.7)	639 (3.2)	272 (6.7)	19 (4.2)
Normal weight	136 335 (51.4)	10 446 (40.6)	7934 (39.6)	1848 (45.9)	219 (48.2)
Overweight	87 906 (33.1)	9788 (38.0)	7781 (38.9)	1321 (32.8)	163 (35.9)
Obese	29 669 (11.2)	4573 (17.8)	3659 (18.3)	589 (14.6)	53 (11.7)
**Waist circumference in cm, median (IQR)**	78.1 (72.0, 84.7)	82.0 (75.5, 89.0)	82.3 (75.9, 89.1)	80.5 (73.2, 87.8)	79.7 (72.6, 86.3)
**Smoking**					
Never smoker	257 531 (97.1)	24 171 (93.9)	18 807 (94.0)	3784 (93.9)	426 (93.8)
Ever smoker	1977 (0.7)	534 (2.1)	385 (1.9)	87 (2.2)	4 (0.9)
Current smoker	5670 (2.1)	1049 (4.1)	821 (4.1)	159 (3.9)	24 (5.3)
**Passive smoking**					
No	102 715 (38.7)	11 918 (46.3)	9391 (46.9)	1673 (41.5)	208 (45.8)
Yes	162 463 (61.3)	13 836 (53.7)	10 622 (53.1)	2357 (58.5)	246 (54.2)
**Drinking**					
Never drinker	253 872 (95.7)	24 581 (95.4)	19 097 (95.4)	3839 (95.3)	428 (94.3)
Ever drinker	2209 (0.8)	298 (1.2)	207 (1.0)	71 (1.8)	6 (1.3)
Current drinker	9097 (3.4)	875 (3.4)	709 (3.5)	120 (3.0)	20 (4.4)
**Physical activity in MET-hours/d, median (IQR)**	17.6 (11.2–29.1)	11.3 (8.4–17.3)	11.4 (8.4–17.0)	12.00 (8.4–20.7)	12.85 (8.4–20.0)
**Marital status**					
Unmarried	25 873 (9.8)	4976 (19.3)	3819 (19.1)	851 (21.1)	62 (13.7)
Married	239 305 (90.2)	20 778 (80.7)	16 194 (80.9)	3179 (78.9)	392 (86.3)
**Parity**					
1	97 716 (36.8)	4433 (17.2)	3646 (18.2)	456 (11.3)	94 (20.7)
2	86 963 (32.8)	7038 (27.3)	5507 (27.5)	961 (23.8)	145 (31.9)
3	45 159 (17.0)	6407 (24.9)	5002 (25.0)	981 (24.3)	109 (24.0)
≥4	35 340 (13.3)	7876 (30.6)	5858 (29.3)	1632 (40.5)	106 (23.3)
**Menopausal status**					
No	123 004 (46.4)	3703 (14.4)	2830 (14.1)	648 (16.1)	96 (21.1)
Yes	142 174 (53.6)	22 051 (85.6)	17 183 (85.9)	3382 (83.9)	358 (78.9)
**Diabetes**					
No	251 199 (94.7)	21 961 (85.3)	17 012 (85.0)	3558 (88.3)	417 (91.9)
Yes	13 979 (5.3)	3793 (14.7)	3001 (15.0)	472 (11.7)	37 (8.1)
**Hypertension**					
No	186 834 (70.5)	10 236 (39.7)	8462 (42.3)	1043 (25.9)	196 (43.2)
Yes	78 344 (29.5)	15 518 (60.3)	11 551 (57.7)	2987 (74.1)	258 (56.8)
**Coronary heart disease**					
No	258 542 (97.5)	23 125 (89.8)	17 922 (89.6)	3 791 (94.1)	429 (94.5)
Yes	6636 (2.5)	2629 (10.2)	2091 (10.4)	239 (5.9)	25 (5.5)
**Oral contraceptive pill usage**					
No	238 139 (89.8)	23 707 (92.1)	18 456 (92.2)	3809 (94.5)	411 (90.5)
Yes	27 039 (10.2)	2047 (7.9)	1557 (7.8)	221 (5.5)	43 (9.5)
**Anticoagulation therapy**					
No	263 289 (99.3)	24 895 (96.7)	19 477 (97.3)	3881 (96.3)	447 (98.5)
Yes	1889 (0.7)	859 (3.3)	536 (2.7)	149 (3.7)	7 (1.5)
**Hypolipidemic therapy**					
No	264 706 (99.8)	25 584 (99.3)	19 899 (99.4)	4002 (99.3)	453 (99.8)
Yes	472 (0.2)	170 (0.7)	114 (0.6)	28 (0.7)	1 (0.2)
**Age at first live birth in years, median (IQR)**	23.0 (21.0–25.0)	23.0 (21.0–25.0)	23.0 (21.0–26.0)	22.0 (20.0–24.0)	23.0 (21.0–25.0)
**Age at first live birth**					
<22	75 896 (28.6)	9127 (35.4)	6656 (33.3)	1982 (49.2)	159 (35.0)
22–24	101 694 (38.3)	8075 (31.4)	6301 (31.5)	1177 (29.2)	166 (36.6)
≥25	87 588 (33.0)	8552 (33.2)	7056 (35.3)	871 (21.6)	129 (28.4)

BMI – body mass index, MET – metabolic equivalent task

*Presented as n (%) unless specified otherwise.

†Total strokes included incident strokes after age at first live birth at baseline and during follow-up, whichever came first. Subtypes of strokes at baseline were ambiguous.

### The associations of AFLB with incident stroke and its subtypes

Compared with parous women whose AFLB was <22 years, parous women with later AFLB had a higher risk of total stroke, with fully adjusted HRs of 1.71 (95% CI = 1.65–1.77) for 22–24 years and 3.37 (95% CI = 3.24–3.51) for ≥25 years (Table S3 in the **Online Supplementary Document**). We also observed increased risks of subtypes of stroke among parous women with AFLB of 22–24 years (ischaemic stroke: aHR = 2.00, 95% CI = 1.92–2.08; intracerebral haemorrhage: aHR = 1.51, 95% CI = 1.39–1.64; subarachnoid haemorrhage: aHR = 1.80, 95% CI = 1.41–2.31) and of ≥25 years (ischaemic stroke: aHR = 4.71, 95% CI = 4.50–4.94; intracerebral haemorrhage: aHR = 3.37, 95% CI = 3.04–3.75; subarachnoid haemorrhage: aHR = 2.92,  95% CI = 2.13–3.99) compared with those giving their first delivery <22 years.

### Urban-rural SES-stratified association of AFLB with incident stroke and its subtypes

**Figure 2:**
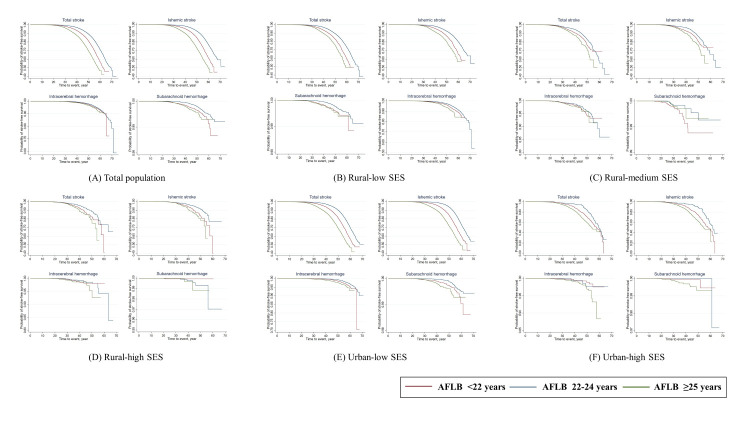
Kaplan-Meier curves by categories of age at first live birth across SES classes. AFLB – age at first live birth, SES – socioeconomic status.

We identified two and three degrees of SES in urban and rural areas according to the distributions of education, occupation, and annual household income. In urban areas, women with low SES were more likely to have their first delivery at <25 years (52.0%). In contrast, those in the high SES class tended to have their first childbirth later, with 78.2% of women whose AFLB was ≥25 years. Among rural residents, the proportions of AFLB ≥25 years ranged from 16.2 to 30.7% across low, medium, and high SES classes (**Figure 2**; Tables S4–6 in the **Online Supplementary Document**).

**Figure 3:**
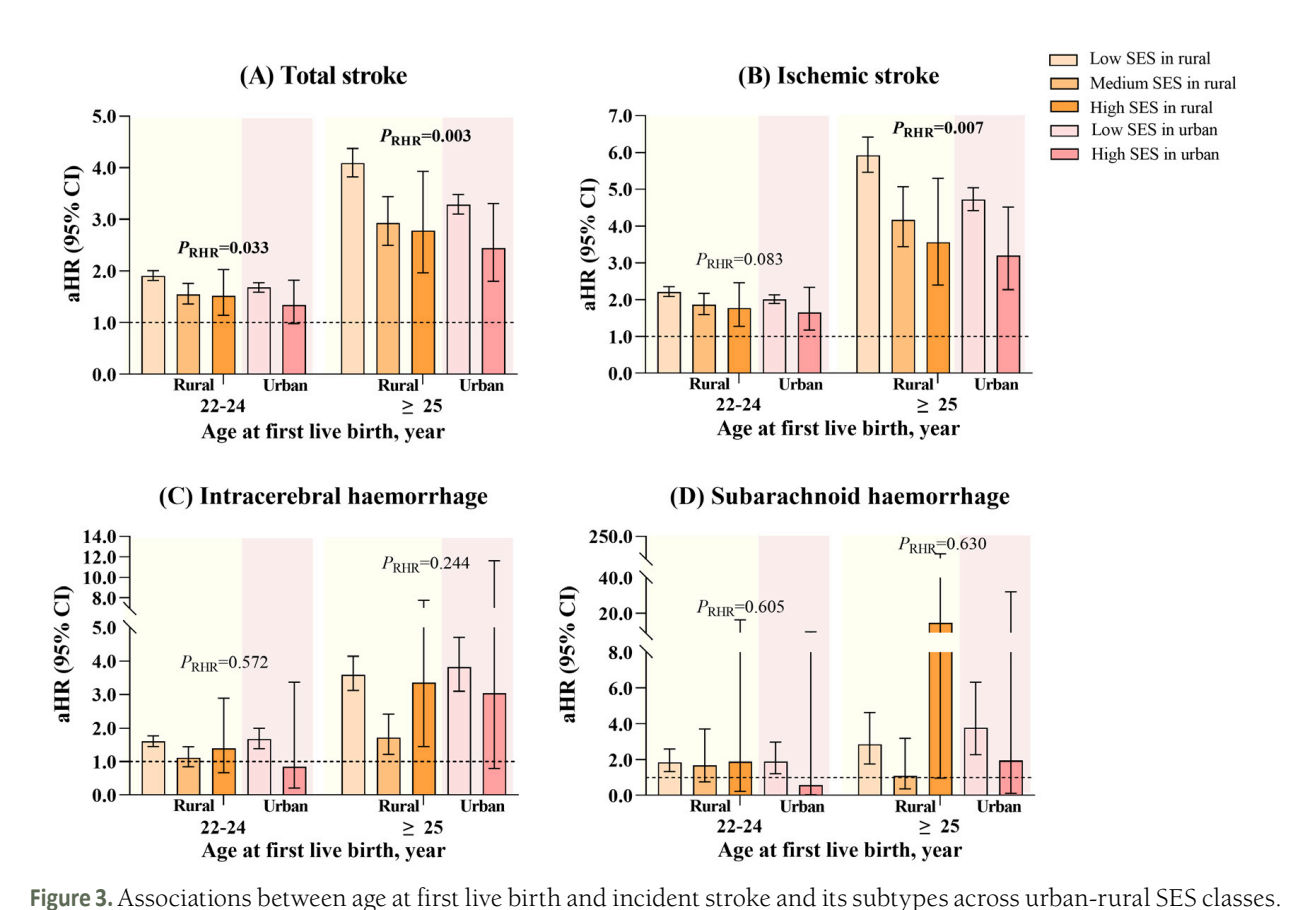
Associations between age at first live birth and incident stroke and its subtypes across urban-rural SES classes. We adjusted the model for age at baseline, residence, body mass index categories, waist circumference, smoking, passive smoking, drinking, physical activity, marital status, diabetes, hypertension, coronary heart disease, oral contraceptive pills usage, anticoagulation therapy, hypolipidemic therapy, menopausal status, and parity. The *P*_RHR_ was for the rural-to-urban RHR in each category of age at first birth. aHR – adjusted hazard ratio, CI – confidence interval, RHR – ratio of hazard ratio, SES – socioeconomic status.

There was a significant interaction between SES classes and AFLB for total stroke and ischaemic stroke (**Table 2** and **Figure 3**). The magnitudes of associations with total stroke were the strongest among rural-low-SES participants, with aHRs of 1.91 (95% CI = 1.82–2.01) for AFLB of 22–24 years and 4.09 (95% CI = 3.82–4.37) for AFLB of ≥25 years. For rural residents in medium and high SES classes, the aHRs for AFLB of 22–24 were 1.55 (95% CI = 1.36–1.76) and 1.52 (95% CI = 1.14–2.03); meanwhile, for AFLB of ≥25 years, the aHRs were 2.93 (95% CI = 2.50–3.44) and 2.78 (95% CI = 1.97–3.93) with total stroke. We found the lowest strength of the association among urban-high-SES ones, as those with AFLB of ≥25 years had 2.44 (95% CI = 1.80–3.31) times greater risks of developing total stroke (*P*-value for interaction = 0.003). Similarly, we observed the highest increased risks of ischaemic stroke among rural-low-SES women with AFLB of ≥25 years (aHR = 5.92; 95% CI = 5.46–6.42) and the lowest increased hazard among urban-high-SES ones (aHR = 3.21; 95% CI = 2.27–4.52) (*P*-value for interaction = 0.011). Regarding intracerebral haemorrhage, we found higher risks among rural women with later AFLB in all three SES classes, except for medium- and high-SES individuals whose AFLB was between 22–24 years. Meanwhile, the association for urban residents was only noticeable among low-SES ones. For subarachnoid haemorrhage, we observed higher risks in rural-low-SES and urban-low-SES women who had the first delivery at >22 years.

**Table 2:** Associations of age at first live birth with incident stroke and its subtypes across urban-rural SES classes

	Rural, aHR (95% CI)*			Urban, aHR (95% CI)*		
**AFLB in years**	**Low (n = 113 945)**	**Medium (n = 39 326)**	**High (n = 8700)**	**Low (n = 114 090)**	**High (n = 14 871)**	***P*-value for interaction**†
**Total stroke**						**0.003**
No of cases	10 050	1657	335	12 386	1326	
<22	ref	ref	ref	ref	ref	
22-24	1.91 (1.82–2.01)	1.55 (1.36–1.76)	1.52 (1.14–2.03)	1.68 (1.59–1.77)	1.34 (0.99–1.82)	
≥25	4.09 (3.82–4.37)	2.93 (2.50–3.44)	2.78 (1.97–3.93)	3.29 (3.10–3.48)	2.44 (1.80–3.31)	
**Ischaemic stroke**						0.011
No of cases	7122	1198	263	10 268	1162	
<22	ref	ref	ref	ref	ref	
22–24	2.21 (2.08–2.35)	1.86 (1.59–2.17)	1.77 (1.28–2.46)	2.01 (1.89–2.13)	1.66 (1.18–2.33)	
≥25	5.92 (5.46–6.42)	4.18 (3.44–5.07)	3.56 (2.40–5.29)	4.72 (4.42–5.04)	3.21 (2.27–4.52)	
**Intracerebral haemorrhage**						0.126
No of cases	2569	348	56	985	72	
<22	ref	ref	ref	ref	ref	
22-24	1.60 (1.45–1.76)	1.11 (0.85–1.45)	1.39 (0.67–2.89)	1.67 (1.39–2.00)	0.85 (0.21–3.37)	
≥25	3.59 (3.12–4.14)	1.71 (1.21–2.42)	3.36 (1.45–7.76)	3.82 (3.10–4.70)	3.04 (0.80–11.61)	
**Subarachnoid haemorrhage**						0.296
No of cases	206	45	9	174	20	
<22	ref	ref	ref	ref	ref	
22–24	1.86 (1.34–2.60)	1.69 (0.77–3.72)	1.88 (0.22–16.22)	1.90 (1.22–2.98)	0.58 (0.04–9.49)	
≥25	2.86 (1.77–4.64)	1.08 (0.37–3.19)	14.77 (0.96–226.22)	3.80 (2.28–6.33)	1.94 (0.12–32.14)	

AFLB – age at first live birth, aHR – adjusted hazard ratio, CI – confidence interval, ref – reference, SES – socioeconomic status

*Model was adjusted for age at baseline, body mass index categories, waist circumference, smoking, passive smoking, drinking, physical activity, marital status, diabetes, hypertension, coronary heart disease, oral contraceptive pills usage, anticoagulation therapy, hypolipidemic therapy, menopausal status, and parity.

†We found significant interaction effects (*P* < 0.05) between AFLB and urban-rural SES classes for total stroke and ischaemic stroke.

### Subgroup analyses within the residence and SES components

In the residence-stratified results, we found stronger magnitudes for total stroke and ischaemic stroke among rural residents (Table S7 in the **Online Supplementary Document**). For instance, the aHRs of ischaemic stroke for AFLB of ≥25 years were 5.43 (95% CI = 5.05–5.85) for rural residents and 4.22 (95% CI = 3.96–4.51) for urban ones, with the rural-to-urban ratio of HR of 1.13 (95% CI = 1.03–1.23). In contrast, we saw stronger associations with intracerebral haemorrhage and subarachnoid haemorrhage within urban residents with older AFLB, with the ratio of HR being non-significant. Moreover, the associations between later AFLB and total and ischaemic stroke were particularly prominent among low-income, less-educated individuals, and labourers (*P*-value for interaction <0.05). Conversely, those who were unemployed, retired, or doing other types of work with AFLB of ≥22 years (*P*-value for interaction = 0.029), and those who had an annual household income in CNY of ≥10 000 with AFLB of ≥25 years (*P*-value for interaction <0.001) had stronger associations with higher risks of intracerebral haemorrhage (Tables S8–10 in the **Online Supplementary Document**).

### Population-attributable fractions across the urban-rural SES classes

**Figure 4:**
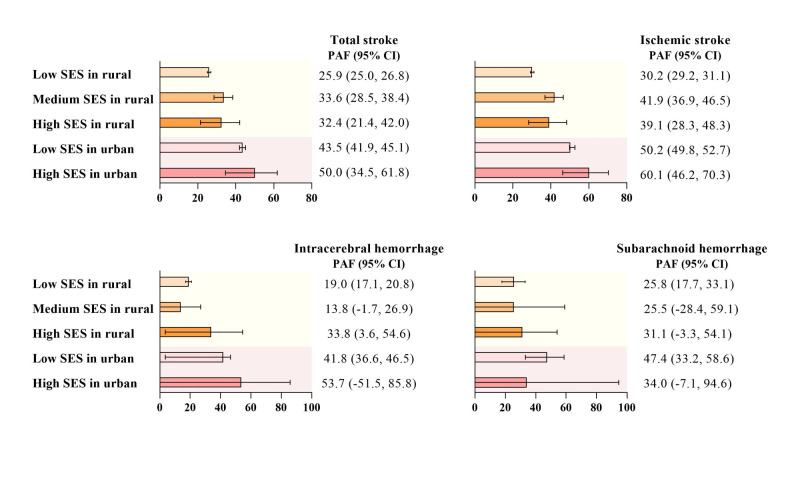
Population attributable fraction of stroke and its subtypes across urban-rural SES classes. PAFs referred to the proportion of stroke cases attributable to the age at first live birth older than 22 years among Chinese parous women and were calculated using ‘punaffc’ package in Stata. AFLB – age at first live birth, CI – confidence interval, PAF – population attributable fraction, SES – socioeconomic status.

The PAFs demonstrated the fractions of the stroke burden attributable to AFLB of ≥22 years among studied Chinese parous women across SES classes (**Figure 4**). We observed the highest PAFs of total stroke (50.0%; 95% CI = 34.5–61.8) and ischaemic stroke (60.1%; 95% CI = 46.2–70.3) for urban-high-SES individuals. Among rural-low-SES women, the PAFs of total stroke and ischaemic stroke were 25.9% and 30.2%, respectively. For intracerebral haemorrhage, the PAFs ranged from 19.0% in rural-low-SES to 41.8% in urban-low-SES. Likewise, we found higher PAF of subarachnoid haemorrhage for urban-low-SES ones (47.4%) compared to rural-low-SES ones (25.8%).

## DISCUSSION

In this large prospective cohort study of the Chinese population, we observed associations of later AFLB, especially AFLB of ≥25 years, with higher risks of stroke and its subtypes among Chinese parous women. However, we also found that these associations might vary depending on different subtypes, maternal SES, and residence, as the strength of the association with ischaemic stroke was stronger among low-SES and rural women, while the highest PAFs of total and ischaemic stroke associated with later AFLB were among urban-high-SES ones.

Previous studies have investigated the associations between AFLB and CVDs among parous women. For example, a case-control study among Japanese women found that those with later age at first pregnancy (≥26 years) were at an increased risk of subarachnoid haemorrhage, which somewhat aligns with our results [32]. In contrast, a retrospective cohort study among Israeli women reported no significant difference in the prevalence of subsequent CVDs between younger parturients with AFLB of 35–39 years and older parturients with AFLB of 40–44 years [33]. Another Mendelian randomisation study based on the UK Biobank showed that AFLB had no relation with stroke in the European population [34]. A cohort study from America reported an inverse, but nonsignificant association between early AFLB and self-reported stroke [35]. These findings in Western countries differed from ours, possibly due to the differences in the study setting, variate measurements, cultural background, and developmental level. Comparatively, our participants had much earlier AFLB than the above studies, with 67.4% having given birth to the first child at the age of <25 years. Moreover, most of our participants gave birth to their first child before 1990, a period when the maternal services and advanced technology were still developing and traditional delivery attendance was undergoing skilled transformation [36]. Notably, the maternal mortality ratio in China was considerably higher in this period, while the coverage and quality of primary health care were lower compared to high-income countries [36,37]. Therefore, our parous participants were more likely to present subsequent health conditions compared to parous women in the UK and USA.

We observed a positive correlation between AFLB and risks of incident ischaemic and haemorrhagic stroke, and demonstrated the urban-rural socioeconomic disparities in the association. This might be due to both physiological and sociological pathways [38]. Physiologically, women would experience significant cardiovascular adaptions during pregnancy and delivery, including reduced peripheral vascular resistance and uterine vasculature. Meanwhile, the adaptive ability of the vascular system in those with older childbearing age might be impaired and fertility-related cardiovascular demands could not be accommodated, which might cause irreversible damage to the ageing mothers [12]. For instance, elevated arterial stiffness due to reduced peripheral vascular resistance might contribute to ischaemic stroke. Additionally, previous studies have proved that pregnancy-related complications were more likely to develop in women with advanced age at birth, which may substantially impact the development of incident CVDs [11,34]. Those with hypertensive disorders of pregnancy would have higher risks of haemorrhagic stroke [4]. Besides, through increased levels of certain hormones (such as oestrogen and progesterone) during pregnancy, a lower AFLB might have a protective influence on the blood-vascular systems and further attenuate the risk of incident stroke [39].

We found that SES classes and residence might have a modifying effect on the associations of AFLB with stroke and its subtypes. Specifically, the association with ischaemic stroke appeared more pronounced among mothers with low SES or living in rural, while rural-low-SES ones faced the highest risks. Low SES comprises lower annual household income, reduced educational attainment, and arduous jobs. Compared to individuals with higher SES, those with lower SES are more likely to adopt poor lifestyle habits [40]. Ischaemic stroke, which arises from an acute interruption of blood flow typically due to embolic or thrombotic occlusion, could be impacted by various endocrine and metabolic factors [41]. Moreover, unhealthy lifestyle behaviours like smoking and drinking could trigger inflammation or endocrine disorders, activate platelets and increase their propensity to form clots, thereby elevating the risk of ischaemic stroke [42]. Besides, socially disadvantaged women might lack health awareness or access to health-promoting services, which could detrimentally influence post-rehabilitation and further health conditions in later life [43]. Additionally, individuals with high SES tend to have great access to health care services and perform better in disease prevention and management, whereas those with low SES may not utilize health care services adequately [44]. Besides, the urban-rural disparity in the AFLB-related stroke risk persisted when adjusting for individual SES variables in our study. Compared to urban dwellers, rural residents tend to face greater health challenges due to less widespread stroke-related health education and inferior quality of stroke care in rural areas [19,45,46]. In China, the inequality between urban and rural regions is significant in various aspects [20]. For instance, the coverage and quality of primary health care services are relatively lower in rural areas than urban areas [37]. This leaves a need to improve health care services for parous women with low SES and investigate tailored strategies in urban and rural areas.

Notably, we found no significant urban-rural disparity among individuals with haemorrhagic stroke. Since different stroke subtypes have diverse aetiologies, pathophysiology, and risk factors, and since the burden varies by region and residence, tailored preventions and interventions should be taken for individuals from various areas or with different stroke subtypes.

In contrast to the results that the highest hazard of ischaemic stroke appeared among rural-low-SES mothers, we found the highest PAFs for urban-high-SES individuals, which may be due to the high probability of delayed childbirth among urban women with higher SES [18]. This suggests that maternal health was not a concern in low SES class and rural residence alone. Consequently, from a policy perspective, promoting various fertility-related prevention strategies could have significant health potential across both urban and rural areas, as well as all levels of SES.

This study has several strengths. First, this is the first prospective study to explore the association of AFLB with stroke among Chinese parous women, as well as the moderating effects of urban-rural SES. Second, we expanded the outcome of the stroke into different subtypes, providing a more comprehensive perspective on the association between AFLB and stroke. Moreover, previous studies only focussed on a single SES factor, such as education level. Since different SES factors reflected different SES domains, a comprehensive variable was needed [47]. To address this, we considered urban-rural disparity and different individual socioeconomic conditions to provide a relatively comprehensive view of the correlation between AFLB and stroke risks. Finally, as a nationwide prospective cohort study of the Chinese population, the CKB provided us with a large and representative sample size from 10 diverse geographical areas across China.

Nevertheless, our study has several limitations. First, the information on AFLB and medical histories was self-reported, which may have led to recall bias. However, previous studies have demonstrated a generally consistent reporting of first-birth events by mothers when compared to hospital records [48]. Likewise, previous studies reported perfect agreements between self-reported and medical records [49]. Second, we only included Chinese parous women, which may restrict the generalisability of our findings. In relation to this, given the variations in reproductive trajectories across different races, there may be racial disparities in the associations between AFLB and the risk of stroke [50]. Third, we classified participants into SES groups based on posterior item probabilities, an approach which might not be completely accurate. However, we based this classification on the best-fit model determined by a series of fit statistics. This approach was further affirmed by our large sample size [27]. Finally, we did not consider some potential covariates, such as prenatal adverse exposure, due to data unavailability. We were therefore unable to explore the potential mediation effects of pregnancy-related complications on the association.

## CONCLUSIONS

In this study, we observed associations between later AFLB and higher risks of incident stroke and its subtypes among Chinese parous women, with significant differences across urban-rural SES classes in ischaemic stroke. Given the increasing prevalence of delayed childbirth in recent years, providing personalised medical advice and services to pregnant women of different ages might have long-term benefits in stroke prevention. Besides, the disparity in stroke risk between rural and urban parous women highlights the importance of enhancing health perception and awareness among all levels of SES and introducing effective policies to ease the financial burden of health care services. Health education campaigns should be implemented to improve the understanding of potential adverse outcomes of childbearing and emphasise the importance of periodic physical examinations, especially among less developed regions. Expanding maternity insurance coverage and improving health care subsidies in low-SES places could also play a role in mitigating the risk of stroke development among parous women.

## Additional material


Online Supplementary Document

## References

[1] Feigin VL Brainin M Norrving B Martins S Sacco RL Hacke W World Stroke Organization (WSO): Global Stroke Fact Sheet 2022. Int J Stroke. 2022;17:18–29. 10.1177/17474930211065917 34986727

[2] Ma Q Li R Wang L Yin P Wang Y Yan C Temporal trend and attributable risk factors of stroke burden in China, 1990-2019: an analysis for the Global Burden of Disease Study 2019. Lancet Public Health. 2021;6:e897–906. 10.1016/S2468-2667(21)00228-0 34838196 PMC9047702

[3] Wu S Wu B Liu M Chen Z Wang W Anderson CS Stroke in China: advances and challenges in epidemiology, prevention, and management. Lancet Neurol. 2019;18:394–405. 10.1016/S1474-4422(18)30500-3 30878104

[4] Campbell BCV Khatri P Stroke. Lancet. 2020;396:129–42. 10.1016/S0140-6736(20)31179-X 32653056

[5] Lain KY Catalano PM Metabolic changes in pregnancy. Clin Obstet Gynecol. 2007;50:938–48. 10.1097/GRF.0b013e31815a5494 17982337

[6] Kohlhepp LM Hollerich G Vo L Hofmann-Kiefer K Rehm M Louwen F [Physiological changes during pregnancy]. Anaesthesist. 2018;67:383–96. German. 10.1007/s00101-018-0437-2 29654495

[7] Challis JR Lockwood CJ Myatt L Norman JE Strauss JF III Petraglia F Inflammation and pregnancy. Reprod Sci. 2009;16:206–15. 10.1177/1933719108329095 19208789

[8] Ouzounian JG Elkayam U Physiologic changes during normal pregnancy and delivery. Cardiol Clin. 2012;30:317–29. 10.1016/j.ccl.2012.05.004 22813360

[9] Rosendaal NTA Pirkle CM Age at first birth and risk of later-life cardiovascular disease: a systematic review of the literature, its limitation, and recommendations for future research. BMC Public Health. 2017;17:627. 10.1186/s12889-017-4519-x 28679414 PMC5498883

[10] Mirowsky J Age at first birth, health, and mortality. J Health Soc Behav. 2005;46:32–50. 10.1177/002214650504600104 15869119

[11] Wolfson C Gemmill A Strobino DM Advanced Maternal Age and Its Association With Cardiovascular Disease in Later Life. Womens Health Issues. 2022;32:219–25. 10.1016/j.whi.2021.12.007 35058125

[12] Cooke CM Davidge ST Advanced maternal age and the impact on maternal and offspring cardiovascular health. Am J Physiol Heart Circ Physiol. 2019;317:H387–H94. 10.1152/ajpheart.00045.2019 31199185

[13] Peters SA Woodward M Women’s reproductive factors and incident cardiovascular disease in the UK Biobank. Heart. 2018;104:1069–75. 10.1136/heartjnl-2017-312289 29335253

[14] Cutler DM, Lleras-Muney A, Vogl T. Socioeconomic status and health: dimensions and mechanisms. Cambridge, Massachusetts, USA: National Bureau of Economic Research; 2008. Available: https://www.nber.org/papers/w14333. Accessed: 2 April 2024.

[15] Lynch J, Kaplan GA. Socioeconomic position. In: Berkamn LF, Kawachi I. Social Epidemiology. New York, USA: Oxford University Press; 2000. p. 13–35.

[16] Lacey RE Kumari M Sacker A McMunn A Age at first birth and cardiovascular risk factors in the 1958 British birth cohort. J Epidemiol Community Health. 2017;71:691–8. 10.1136/jech-2016-208196 28270503 PMC5485753

[17] Umberson D Crosnoe R Reczek C Social Relationships and Health Behavior Across Life Course. Annu Rev Sociol. 2010;36:139–57. 10.1146/annurev-soc-070308-120011 21921974 PMC3171805

[18] Schummers L Hutcheon JA Hacker MR VanderWeele TJ Williams PL McElrath TF Absolute risks of obstetric outcomes by maternal age at first birth: a population-based cohort. Epidemiology. 2018;29:379–87. 10.1097/EDE.0000000000000818 29517506 PMC5922259

[19] Dwyer M Rehman S Ottavi T Stankovich J Gall S Peterson G Urban-rural differences in the care and outcomes of acute stroke patients: Systematic review. J Neurol Sci. 2019;397:63–74. 10.1016/j.jns.2018.12.021 30594105

[20] Li H Zeng Y Gan L Tuersun Y Yang J Liu J Urban-rural disparities in the healthy ageing trajectory in China: a population-based study. BMC Public Health. 2022;22:1406. 10.1186/s12889-022-13757-x 35870914 PMC9308310

[21] Chen Z Lee L Chen J Collins R Wu F Guo Y Cohort profile: the Kadoorie Study of Chronic Disease in China (KSCDC). Int J Epidemiol. 2005;34:1243–9. 10.1093/ije/dyi174 16131516

[22] Chen Z Chen J Collins R Guo Y Peto R Wu F China Kadoorie Biobank of 0.5 million people: survey methods, baseline characteristics and long-term follow-up. Int J Epidemiol. 2011;40:1652–66. 10.1093/ije/dyr120 22158673 PMC3235021

[23] Yang CY Chang CC Kuo HW Chiu HF Parity and risk of death from subarachnoid hemorrhage in women: evidence from a cohort in Taiwan. Neurology. 2006;67:514–5. 10.1212/01.wnl.0000227938.06750.ec 16894119

[24] Gao M Lv J Yu C Guo Y Bian Z Yang R Metabolically healthy obesity, transition to unhealthy metabolic status, and vascular disease in Chinese adults: A cohort study. PLoS Med. 2020;17:e1003351. 10.1371/journal.pmed.1003351 33125374 PMC7598496

[25] Lanza ST, Dziak JJ, Huang L, Wagner AT, Collins LM. LCA Stata plugin users’ guide (Version 1.2). State College, Pennsylvania, USA: The Methodology Center, Penn State; 2015. Available: https://bpb-us-e1.wpmucdn.com/sites.psu.edu/dist/f/84470/files/2019/03/Stata-LCA-Plugin-v1.2c-2e00dl9.pdf. Accessed: 2 April 2024.

[26] Afroz N Kabir E Alam K A latent class analysis of the socio-demographic factors and associations with mental and behavioral disorders among Australian children and adolescents. PLoS One. 2023;18:e0285940. 10.1371/journal.pone.0285940 37200385 PMC10194994

[27] Sinha P Calfee CS Delucchi KL Practitioner’s Guide to Latent Class Analysis: Methodological Considerations and Common Pitfalls. Crit Care Med. 2021;49:e63–79. 10.1097/CCM.0000000000004710 33165028 PMC7746621

[28] Lowthian E Page N Melendez-Torres GJ Murphy S Hewitt G Moore G Using Latent Class Analysis to Explore Complex Associations Between Socioeconomic Status and Adolescent Health and Well-Being. J Adolesc Health. 2021;69:774–81. 10.1016/j.jadohealth.2021.06.013 34275658 PMC9225957

[29] Woodward M Rationale and tutorial for analysing and reporting sex differences in cardiovascular associations. Heart. 2019;105:1701–8. 10.1136/heartjnl-2019-315299 31371439 PMC6855792

[30] Lee M Whitsel E Avery C Hughes TM Griswold ME Sedaghat S Variation in Population Attributable Fraction of Dementia Associated With Potentially Modifiable Risk Factors by Race and Ethnicity in the US. JAMA Netw Open. 2022;5:e2219672. 10.1001/jamanetworkopen.2022.19672 35793088 PMC9260480

[31] Newson RB Attributable and Unattributable Risks and Fractions and other Scenario Comparisons. The Stata Journal: Promoting communications on statistics and Stata. 2013;13:672–98. 10.1177/1536867X1301300402

[32] Okamoto K Horisawa R Kawamura T Asai A Ogino M Takagi T Menstrual and reproductive factors for subarachnoid hemorrhage risk in women: a case-control study in nagoya, Japan. Stroke. 2001;32:2841–4. 10.1161/hs1201.099383 11739984

[33] Feldman B Orbach-Zinger S Leventer-Roberts M Hoshen M Dagan N Balicer R Maternal age and cardiovascular and metabolic disease outcomes: a retrospective cohort study using data from population-based electronic medical records. J Matern Fetal Neonatal Med. 2020;33:1853–60. 10.1080/14767058.2018.1531844 30278799

[34] Chen M Wang Z Xu H Chen X Teng P Ma L Genetic liability to age at first sex and birth in relation to cardiovascular diseases: a Mendelian randomization study. BMC Med Genomics. 2023;16:75. 10.1186/s12920-023-01496-w 37024926 PMC10080931

[35] Henretta JC Early childbearing, marital status, and women’s health and mortality after age 50. J Health Soc Behav. 2007;48:254–66. 10.1177/002214650704800304 17982867

[36] Qiao J Wang Y Li X Jiang F Zhang Y Ma J A Lancet Commission on 70 years of women’s reproductive, maternal, newborn, child, and adolescent health in China. Lancet. 2021;397:2497–536. 10.1016/S0140-6736(20)32708-2 34043953

[37] Li X Lu J Hu S Cheng KK De Maeseneer J Meng Q The primary health-care system in China. Lancet. 2017;390:2584–94. 10.1016/S0140-6736(17)33109-4 29231837

[38] Wang Z Lu J Weng W Zhang L Zhang J Women’s reproductive traits and ischemic stroke: a two-sample Mendelian randomization study. Ann Clin Transl Neurol. 2023;10:70–83. 10.1002/acn3.51702 36398399 PMC9852390

[39] Hou L Li S Zhu S Yi Q Liu W Wu Y Lifetime Cumulative Effect of Reproductive Factors on Stroke and Its Subtypes in Postmenopausal Chinese Women: A Prospective Cohort Study. Neurology. 2023;100:e1574–86. 10.1212/WNL.0000000000206863 36725338 PMC10103112

[40] The Lancet Public Health Education: a neglected social determinant of health. Lancet Public Health. 2020;5:e361. 10.1016/S2468-2667(20)30144-4 32619534 PMC7326385

[41] Diener H-C Hankey GJ Primary and Secondary Prevention of Ischemic Stroke and Cerebral Hemorrhage: JACC Focus Seminar. J Am Coll Cardiol. 2020;75:1804–18. 10.1016/j.jacc.2019.12.072 32299593

[42] Mukamal KJ The effects of smoking and drinking on cardiovascular disease and risk factors. Alcohol Res Health. 2006;29:199–202. 17373409 PMC6527044

[43] Petrovic D de Mestral C Bochud M Bartley M Kivimäki M Vineis P The contribution of health behaviors to socioeconomic inequalities in health: A systematic review. Prev Med. 2018;113:15–31. 10.1016/j.ypmed.2018.05.003 29752959

[44] Chen L Tan Y Yu C Guo Y Pei P Yang L Educational disparities in ischaemic heart disease among 0.5 million Chinese adults: a cohort study. J Epidemiol Community Health. 2021;75:1033–43. 10.1136/jech-2020-216314 33782052 PMC8515104

[45] Mullen MT Judd S Howard VJ Kasner SE Branas CC Albright KC Disparities in evaluation at certified primary stroke centers: reasons for geographic and racial differences in stroke. Stroke. 2013;44:1930–5. 10.1161/STROKEAHA.111.000162 23640827 PMC3747032

[46] Khan JA Casper M Asimos AW Clarkson L Enright D Fehrs LJ Geographic and sociodemographic disparities in drive times to Joint Commission-certified primary stroke centers in North Carolina, South Carolina, and Georgia. Prev Chronic Dis. 2011;8:A79. 21672403 PMC3136973

[47] Zhang YB Chen C Pan XF Guo J Li Y Franco OH Associations of healthy lifestyle and socioeconomic status with mortality and incident cardiovascular disease: two prospective cohort studies. BMJ. 2021;373:n604. 10.1136/bmj.n604 33853828 PMC8044922

[48] Ebrahimoff M Agreement between maternal recall of distant first birth events with hospital birth records: A cohort study. Research Square [preprint]. 2021. . Accessed: 2 April 2024. 10.21203/rs.3.rs-951334/v1

[49] Zhu K McKnight B Stergachis A Daling JR Levine RS Comparison of self-report data and medical records data: results from a case-control study on prostate cancer. Int J Epidemiol. 1999;28:409–17. 10.1093/ije/28.3.409 10405842

[50] Gold EB Bromberger J Crawford S Samuels S Greendale GA Harlow SD Factors associated with age at natural menopause in a multiethnic sample of midlife women. Am J Epidemiol. 2001;153:865–74. 10.1093/aje/153.9.865 11323317

