# Supplementary material for: Socioeconomic disparities in the association of age at first live birth with incident stroke among Chinese parous women: A prospective cohort study
Source: J Glob Health. 2024 Apr 8;14:04091. doi: 10.7189/jogh.14.04091 (PMC11000532; doi:10.7189/jogh.14.04091)
Supplement: Online Supplementary Document [file jogh-14-04091-s001.pdf]

Legends

Table S1. Test of goodness of fit of latent class models..2
Table S2. Incidence rate of stroke and its subtypes by age at first live birth 3
Table S3. Associations of age at first live birth with incident stroke and its subtypes.......................4
Table S4. Baseline characteristics of included participants among urban residents according to SES classes 5
Table S5. Baseline characteristics of included participants among rural residents according to SES classes 7
Table S6. Incidence rate of stroke and its subtypes by age at first live birth across urban-rural SES classes 10
Table S7. Association of age at first live birth with incident stroke and its subtypes by residence 11
Table S8. Association of age at first live birth with incident stroke and its subtypes by annual household income 12
Table S9. Association of age at first live birth with incident stroke and its subtypes by education 13
Table S10. Association of age at first live birth with incident stroke and its subtypes by occupation 14

Table S1. Test of goodness of fit of latent class models

Group	Loglik	AIC	BIC	Entropy	Latent class marginal probabilities (%)				
					Class 1	Class 2	Class 3	Class 4	Class 5
**Rural**									
1	-443308.7	886635.4	886725.3	1.000	100.0				
2	-434691.2	869420.5	869610.4	0.904	4.6	95.4			
**3**	**-432258.8**	**865157.1**	**865446.9**	**0.888**	**5.4**	**24.3**	**70.3**		
4	-430068.4	860214.8	860604.6	0.765	1.2	4.9	23.5	70.4	
5	-429933.1	859964.3	860454.1	0.521	1.2	3.4	19.5	32.0	44.0
**Urban**									
1	-478671.3	957360.6	957448.5	1.000	100.0				
**2**	**-459981.1**	**920000.2**	**920185.8**	**0.859**	**11.5**	**88.5**			
3	-457019.7	914097.4	914380.6	0.547	11.1	34.1	54.9		
4	-456483.6	913045.2	913426.2	0.494	11.0	13.4	26.5	49.2	
5	-456260.1	912618.2	913096.7	0.546	4.2	3.2	14.1	29.2	43.3

Notes: AIC, Akaike information criterion. BIC, Bayesian information criterion.

Table S2. Incidence rate of stroke and its subtypes by age at first live birth

	Total stroke	Ischemic stroke	Intracerebral hemorrhage	Subarachnoid hemorrhage
Cases (n)	25,754	20,013	3,406	454
Age at first live birth				
<22	2455.97	1781.55	527.80	42.31
22-24	2098.18	1629.86	302.95	42.70
≥25	2746.87	2253.61	276.17	40.88

Notes: Incidence rate was calculated in 1,000,000 person-years.

Table S3. Associations of age at first live birth with incident stroke and its subtypes

AFLB, years	Model 1	Model 2	Model 3	Model 4
	aHR (95% CI)			
**Total stroke**				
<22	reference	reference	reference	reference
22-24	**1.82 (1.76, 1.88)**	**1.80 (1.74, 1.86)**	**1.76 (1.70, 1.82)**	**1.71 (1.65, 1.77)**
≥25	**4.01 (3.87, 4.16)**	**3.86 (3.71, 4.01)**	**3.68 (3.54, 3.83)**	**3.37 (3.24, 3.51)**
**Ischemic stroke**				
<22	reference	reference	reference	reference
22-24	**2.21 (2.13, 2.30)**	**2.14 (2.06, 2.23)**	**2.06 (1.98, 2.15)**	**2.00 (1.92, 2.08)**
≥25	**6.11 (5.85, 6.37)**	**5.61 (5.36, 5.87)**	**5.18 (4.95, 5.43)**	**4.71 (4.50, 4.94)**
**Intracerebral hemorrhage**				
<22	reference	reference	reference	reference
22-24	**1.24 (1.14, 1.34)**	**1.42 (1.31, 1.53)**	**1.53 (1.41, 1.66)**	**1.51 (1.39, 1.64)**
≥25	**2.18 (1.98, 2.41)**	**2.89 (2.61, 3.20)**	**3.49 (3.14, 3.86)**	**3.37 (3.04, 3.75)**
**Subarachnoid hemorrhage**				
<22	reference	reference	reference	reference
22-24	**1.89 (1.48, 2.40)**	**1.83 (1.43, 2.34)**	**1.85 (1.44, 2.37)**	**1.80 (1.41, 2.31)**
≥25	**3.20 (2.41, 4.25)**	**2.97 (2.20, 4.02)**	**3.07 (2.26, 4.18)**	**2.92 (2.13, 3.99)**

Notes: aHR, adjusted hazard ratio. CI, confidence interval. AFLB, age at first live birth. Model 1 was adjusted for age at baseline. Model 2 was further adjusted for body mass index categories, waist circumference, smoking, passive smoking, drinking, physical activity, marital status, diabetes, hypertension, coronary heart disease, oral contraceptive pills usage, anticoagulation therapy, hypolipidemic therapy, menopausal status, and parity. Model 3 was further adjusted for residence based on Model 2. Model 4 was further adjusted for education, occupation, and annual household income based on Model 3.

Table S4. Baseline characteristics of included participants among urban residents according to SES classes

Baseline characteristics	Socioeconomic status in urban area		*P* value
	Low (N=114,090)	High (N=14,871)	
Age, year	52.66 (44.54, 60.98)	45.28 (39.77, 53.01)	<0.001
Education, n (%)			<0.001
Primary school and below	48297 (42.3)	0 (0.0)	
Middle school	38663 (33.9)	348 (2.3)	
High school and above	27130 (23.7)	14523 (97.7)	
Occupation, n (%)			<0.001
Unemployed, retired or others	74322 (65.1)	3480 (23.4)	
Farmer or worker	25204 (22.1)	997 (6.7)	
Sales, self-employed, manager or professional	14564 (12.8)	10394 (69.9)	
Annual household income, n (%)			<0.001
<10,000	19399 (17.0)	192 (1.3)	
10,000-34,999	72203 (63.2)	7239 (48.7)	
≥35000	22488 (19.7)	7440 (50.0)	
Body mass index, kg/m²	24.10 (21.90, 26.50)	23.20 (21.40, 25.30)	<0.001
Body mass index status, n (%)			<0.001
Underweight	3537 (3.1)	422 (2.8)	
Normal weight	51432 (45.1)	8505 (57.2)	
Overweight	42098 (36.9)	4737 (31.9)	
Obesity	17023 (14.9)	1207 (8.1)	
Waist circumference, cm	80.00 (73.50, 86.50)	76.40 (71.00, 82.10)	<0.001
Smoking, n (%)			<0.001
Never smoker	110601 (96.9)	14751 (99.2)	
Ever smoker	1007 (0.9)	31 (0.2)	
Current smoker	2482 (2.2)	89 (0.6)	
Passive smoking, n (%)			<0.001
No	52415 (45.9)	7127 (47.9)	
Yes	61675 (54.1)	7744 (52.1)	
Drinking, n (%)			<0.001
Never drinker	109705 (96.2)	13527 (91.0)	
Ever drinker	564 (0.5)	98 (0.7)	
Current drinker	3821 (3.3)	1246 (8.4)	
Physical activity, MET-hours/day	14.00 (8.76, 23.69)	17.46 (13.80, 21.73)	<0.001
Marital status			<0.001
Unmarried	15940 (14.0)	1276 (8.6)	
Married	98150 (86.0)	13595 (91.4)	
Parity, n (%)			<0.001
1	55427 (48.6)	11962 (80.4)	
2	28717 (25.2)	2174 (14.6)	
3	16444 (14.4)	607 (4.1)	
≥4	13502 (11.8)	128 (0.9)	
Menopausal status, n (%)			<0.001
No	42319 (37.1)	9205 (61.9)	

Baseline characteristics	Socioeconomic status in urban area		*P* value
	Low (N=114,090)	High (N=14,871)	
Yes	71771 (62.9)	5666 (38.1)	
Diabetes, n (%)			<0.001
No	104514 (91.6)	14153 (95.2)	
Yes	9576 (8.4)	718 (4.8)	
Hypertension, n (%)			<0.001
No	76967 (67.5)	12257 (82.4)	
Yes	37123 (32.5)	2614 (17.6)	
Coronary heart disease, n (%)			0.750
No	108292 (94.9)	14106 (94.9)	
Yes	5798 (5.1)	765 (5.1)	
Oral contraceptive pills usage, n (%)			<0.001
No	100686 (88.3)	13513 (90.9)	
Yes	13404 (11.7)	1358 (9.1)	
Anticoagulation therapy, n (%)			0.001
No	112940 (99.0)	14762 (99.3)	
Yes	1150 (1.0)	109 (0.7)	
Hypolipidemic therapy, n (%)			0.150
No	113902 (99.8)	14854 (99.9)	
Yes	188 (0.2)	17 (0.1)	
Age at first live birth, year	24.00 (22.00, 26.00)	26.00 (25.00, 28.00)	<0.001
Age at first live birth, n (%)			<0.001
<22	19876 (17.4)	316 (2.1)	
22-24	39429 (34.6)	2920 (19.6)	
≥25	54785 (48.0)	11635 (78.2)	

Notes: Values are presented as number (N) with percent (%) or medians with interquartile ranges (IQRs). SES, socioeconomic status. MET, Metabolic equivalent of task.

Table S5. Baseline characteristics of included participants among rural residents according to SES classes

Characteristics	Socioeconomic status in rural area			*P* value
	Low (N=113,945)	Middle (N=39,326)	High (N=8,700)	
Age, year	53.20 (45.26, 59.81)	42.48 (39.10, 46.88)	43.72 (39.65, 50.17)	<0.001
Education, n (%)				<0.001
Primary school and below	113945 (100.0)	0 (0.0)	2445 (28.1)	
Middle school	0 (0.0)	32411 (82.4)	2932 (33.7)	
High school and above	0 (0.0)	6915 (17.6)	3323 (38.2)	
Occupation, n (%)				<0.001
Unemployed, retired or others	24942 (21.9)	4336 (11.0)	1792 (20.6)	
Farmer or worker	88742 (77.9)	34823 (88.5)	84 (1.0)	
Sales, self-employed, manager or professional	261 (0.2)	167 (0.4)	6824 (78.4)	
Annual household income, n (%)				<0.001
<10,000	51748 (45.4)	14409 (36.6)	89 (1.0)	
10,000-34,999	50631 (44.4)	22585 (57.4)	4207 (48.3)	
≥35000	11566 (10.2)	2332 (5.9)	4404 (50.6)	
Body mass index, kg/m²	23.20 (21.00, 25.60)	23.30 (21.30, 25.60)	23.10 (21.10, 25.30)	<0.001
Body mass index status, n (%)				<0.001
Underweight	6505 (5.7)	1424 (3.6)	327 (3.8)	
Normal weight	60486 (53.1)	21397 (54.4)	4961 (57.0)	
Overweight	35538 (31.2)	12627 (32.1)	2694 (31.0)	
Obesity	11416 (10.0)	3878 (9.9)	718 (8.3)	
Waist circumference, cm	78.10 (71.70, 85.00)	76.80 (71.20, 83.10)	76.90 (71.30, 82.70)	<0.001
Smoking, n (%)				<0.001
Never smoker	108938 (95.6)	38812 (98.7)	8600 (98.9)	
Ever smoker	1362 (1.2)	97 (0.2)	14 (0.2)	

Characteristics	Socioeconomic status in rural area			*P* value
	**Low (N=113,945)**	**Middle (N=39,326)**	**High (N=8,700)**	
Current smoker	3645 (3.2)	417 (1.1)	86 (1.0)	
Passive smoking, n (%)				<0.001
No	41352 (36.3)	11137 (28.3)	2602 (29.9)	
Yes	72593 (63.7)	28189 (71.7)	6098 (70.1)	
Drinking, n (%)				<0.001
Never drinker	108909 (95.6)	37956 (96.5)	8356 (96.0)	
Ever drinker	1500 (1.3)	277 (0.7)	68 (0.8)	
Current drinker	3536 (3.1)	1093 (2.8)	276 (3.2)	
Physical activity, MET-hours/day	19.74 (11.20, 31.03)	19.01 (11.73, 30.04)	21.97 (14.77, 33.34)	<0.001
Marital status				<0.001
Unmarried	12144 (10.7)	1170 (3.0)	319 (3.7)	
Married	101801 (89.3)	38156 (97.0)	8381 (96.3)	
Parity, n (%)				<0.001
1	17667 (15.5)	13190 (33.5)	3903 (44.9)	
2	41496 (36.4)	18028 (45.8)	3586 (41.2)	
3	27660 (24.3)	5956 (15.1)	899 (10.3)	
≥4	27122 (23.8)	2152 (5.5)	312 (3.6)	
Menopausal status, n (%)				<0.001
No	38375 (33.7)	30794 (78.3)	6014 (69.1)	
Yes	75570 (66.3)	8532 (21.7)	2686 (30.9)	
Diabetes, n (%)				<0.001
No	107820 (94.6)	38308 (97.4)	8365 (96.1)	
Yes	6125 (5.4)	1018 (2.6)	335 (3.9)	
Hypertension, n (%)				<0.001
No	70194 (61.6)	31044 (78.9)	6608 (76.0)	
Yes	43751 (38.4)	8282 (21.1)	2092 (24.0)	
Coronary heart disease, n (%)				0.75

Characteristics	Socioeconomic status in rural area			*P* value
	**Low (N=113,945)**	**Middle (N=39,326)**	**High (N=8,700)**	
No	111707 (98.0)	38981 (99.1)	8581 (98.6)	
Yes	2238 (2.0)	345 (0.9)	119 (1.4)	
Oral contraceptive pills usage, n (%)				<0.001
No	103938 (91.2)	36445 (92.7)	7264 (83.5)	
Yes	10007 (8.8)	2881 (7.3)	1436 (16.5)	
Anticoagulation therapy, n (%)				0.001
No	112811 (99.0)	39023 (99.2)	8648 (99.4)	
Yes	1134 (1.0)	303 (0.8)	52 (0.6)	
Hypolipidemic therapy, n (%)				0.15
No	113603 (99.7)	39261 (99.8)	8670 (99.7)	
Yes	342 (0.3)	65 (0.2)	30 (0.3)	
Age at first live birth, year	22.00 (20.00, 24.00)	23.00 (22.00, 24.00)	23.00 (22.00, 25.00)	<0.001
Age at first live birth, n (%)				<0.001
<22	53232 (46.7)	9478 (24.1)	2121 (24.4)	
22-24	42270 (37.1)	21240 (54.0)	3910 (44.9)	
≥25	18443 (16.2)	8608 (21.9)	2669 (30.7)	

Notes: Values are presented as numbers (N) with percent (%) or medians with interquartile ranges (IQRs). SES, socioeconomic status. MET, Metabolic equivalent of task.

Table S6. Incidence rate of stroke and its subtypes by age at first live birth across urban-rural SES classes

Age at first live birth, year	Rural			Urban	
	**Low SES**	**Medium SES**	**High SES**	**Low SES**	**High SES**
**Total stroke**					
<22	2309.7	1440.6	1330.1	3216.8	2309.7
22-24	1993.6	1254.9	1130.3	2606.4	1993.6
≥25	2386.0	1744.9	1285.7	3009.8	2386.0
**Ischemic stroke**					
<22	1590.0	1009.9	1001.0	2546.6	3911.0
22-24	1423.2	908.7	904.2	2157.4	2441.1
≥25	1794.7	1285.0	1018.5	2520.5	2642.8
**Intracerebral hemorrhage**					
<22	652.9	348.6	220.2	303.8	226.6
22-24	446.2	256.6	165.5	198.2	95.6
≥25	496.7	318.1	250.6	213.7	178.0
**Subarachnoid hemorrhage**					
<22	43.0	28.8	64.7	42.6	75.5
22-24	47.7	46.2	16.5	39.5	21.2
≥25	45.4	28.5	26.4	39.8	50.4

Notes: Incidence rate was calculated in 1,000,000 person-years. SES, socioeconomic status.

Table S7. Association of age at first live birth with incident stroke and its subtypes by residence

AFLB, years	Rural (N=161,971)	Urban (N=128,961)	Rural-to-urban RHR	P_{RHR}
	aHR (95% CI)			
**Total stroke**				
No. of cases	8,497	13,819		
<22	reference	reference	reference	
22-24	**1.84 (1.76, 1.92)**	**1.61 (1.53, 1.69)**	**1.07 (1.01, 1.15)**	**0.033**
≥25	**3.81 (3.58, 4.04)**	**2.97 (2.80, 3.14)**	**1.12 (1.04, 1.21)**	**0.003**
**Ischemic stroke**				
No. of cases	6,242	10,939		
<22	reference	reference	reference	
22-24	**2.14 (2.02, 2.26)**	**1.92 (1.81, 2.04)**	1.07 (0.99, 1.15)	0.083
≥25	**5.43 (5.05, 5.85)**	**4.22 (3.96, 4.51)**	**1.13 (1.03, 1.23)**	**0.007**
**Intracerebral hemorrhage**				
No. of cases	1,841	1,850		
<22	reference	reference	reference	
22-24	**1.52 (1.38, 1.66)**	**1.61 (1.34, 1.93)**	0.95 (0.78, 1.15)	0.572
≥25	**3.20 (2.81, 3.64)**	**3.69 (3.00, 4.54)**	0.88 (0.71, 1.09)	0.244
**Subarachnoid hemorrhage**				
No. of cases	138	260		
<22	reference	reference	reference	
22-24	**1.83 (1.35, 2.48)**	**1.76 (1.13, 2.75)**	1.14 (0.69, 1.90)	0.605
≥25	**2.33 (1.51, 3.61)**	**3.56 (2.15, 5.90)**	0.86 (0.47, 1.57)	0.630

Notes: aHR, adjusted hazard ratio. CI, confidence interval. RHR, ratio of hazard ratio. Model was adjusted for age at baseline, body mass index categories, waist circumference, smoking, passive smoking, drinking, physical activity, marital status, diabetes, hypertension, coronary heart disease, oral contraceptive pills usage, anticoagulation therapy, hypolipidemic therapy, menopausal status, parity, education, occupation, and annual household income.

Table S8. Association of age at first live birth with incident stroke and its subtypes by annual household income

AFLB, years	Annual household income <CNY 10,000 (N=85,837)	Annual household income CNY 10,000-34,999 (N=156,865)	Annual household income ≥CNY 35,000 (N=48,230)	*P* for interaction
	aHR (95% CI)			
**Total stroke**				
No. of cases	8,497	13,819	3,438	
<22	reference	reference	reference	
22-24	**1.74 (1.65, 1.84)**	**1.69 (1.61, 1.77)**	**1.58 (1.42, 1.76)**	**<0.001**
≥25	**3.74 (3.48, 4.01)**	**3.14 (2.97, 3.32)**	**3.06 (2.72, 3.45)**	
**Ischemic stroke**				
No. of cases	6,242	10,939	2,832	
<22	reference	reference	reference	
22-24	**2.08 (1.95, 2.22)**	**1.95 (1.85, 2.06)**	**1.84 (1.63, 2.07)**	**<0.001**
≥25	**5.66 (5.20, 6.15)**	**4.27 (4.00, 4.55)**	**4.14 (3.62, 4.74)**	
**Intracerebral hemorrhage**				
No. of cases	1,841	1,850	339	
<22	reference	reference	reference	
22-24	**1.58 (1.41, 1.78)**	**1.48 (1.31, 1.67)**	1.23 (0.90, 1.70)	**<0.001**
≥25	**3.27 (2.77, 3.86)**	**3.24 (2.78, 3.76)**	**3.51 (2.44, 5.07)**	
**Subarachnoid hemorrhage**				
No. of cases	138	260	56	
<22	reference	reference	reference	
22-24	**1.70 (1.11, 2.60)**	**2.06 (1.47, 2.89)**	1.09 (0.50, 2.36)	0.740
≥25	**2.79 (1.56, 4.98)**	**3.17 (2.10, 4.80)**	1.94 (0.77, 4.92)	

Notes: aHR, adjusted hazard ratio. CI, confidence interval. AFLB, age at first live birth. Model was adjusted for age at baseline, residence, body mass index categories, waist circumference, smoking, passive smoking, drinking, physical activity, marital status, diabetes, hypertension, coronary heart disease, oral contraceptive pills usage, anticoagulation therapy, hypolipidemic therapy, menopausal status, parity, education, and occupation. Significant interaction effects (*P interaction* <0.05) between AFLB and annual household income were found for total stroke, ischemic stroke, and intracerebral hemorrhage.

Table S9. Association of age at first live birth with incident stroke and its subtypes by education

AFLB, years	Primary school and below (N=164,687)	Middle school (N=74,354)	High school and above (N=51,891)	*P* for interaction
	aHR (95% CI)			
**Total stroke**				
No. of cases	16,267	5,236	4,251	
<22	reference	reference	reference	
22-24	**1.79 (1.72, 1.86)**	**1.45 (1.33, 1.59)**	**1.62 (1.40, 1.86)**	**<0.001**
≥25	**3.63 (3.46, 3.82)**	**2.70 (2.45, 2.97)**	**2.70 (2.34, 3.12)**	
**Ischemic stroke**				
No. of cases	12,220	4,203	3,590	
<22	reference	reference	reference	
22-24	**2.07 (1.98, 2.17)**	**1.74 (1.57, 1.93)**	**1.98 (1.69, 2.33)**	**0.003**
≥25	**5.19 (4.90, 5.50)**	**3.84 (3.43, 4.30)**	**3.67 (3.10, 4.33)**	
**Intracerebral hemorrhage**				
No. of cases	3,156	549	325	
<22	reference	reference	reference	
22-24	**1.58 (1.44, 1.72)**	**1.15 (0.90, 1.46)**	1.24 (0.82, 1.88)	0.514
≥25	**3.66 (3.24, 4.13)**	**2.28 (1.72, 3.01)**	**2.13 (1.37, 3.31)**	
**Subarachnoid hemorrhage**				
No. of cases	296	101	57	
<22	reference	reference	reference	
22-24	**1.92 (1.45, 2.53)**	**2.14 (1.07, 4.28)**	0.78 (0.27, 2.25)	0.345
≥25	**2.97 (2.01, 4.40)**	**2.84 (1.31, 6.15)**	1.72 (0.57, 5.19)	

Notes: aHR, adjusted hazard ratio. CI, confidence interval. AFLB, age at first live birth. Model was adjusted for age at baseline, residence, body mass index categories, waist circumference, smoking, passive smoking, drinking, physical activity, marital status, diabetes, hypertension, coronary heart disease, oral contraceptive pills usage, anticoagulation therapy, hypolipidemic therapy, menopausal status, parity, occupation, and annual household income. Significant interaction effects (*P interaction* <0.05) between AFLB and education were found for total stroke and ischemic stroke.

Table S10. Association of age at first live birth with incident stroke and its subtypes by occupation

AFLB, years	Unemployed, retired or others (N=108,872)	Farmer or worker (N=149,850)	Sales, self-employed, manager or professional (N=32,210)	*P* for interaction
	aHR (95% CI)			
**Total stroke**				
No. of cases	16,075	8,265	1,414	
<22	reference	reference	reference	
22-24	**1.63 (1.56, 1.70)**	**1.90 (1.79, 2.01)**	**1.50 (1.20, 1.86)**	**<0.001**
≥25	**3.02 (2.87, 3.18)**	**4.02 (3.73, 4.32)**	**3.13 (2.48, 3.96)**	
**Ischemic stroke**				
No. of cases	12,660	6,132	1,221	
<22	reference	reference	reference	
22-24	**1.91 (1.82, 2.01)**	**2.19 (2.05, 2.35)**	**1.72 (1.35, 2.20)**	**<0.001**
≥25	**4.36 (4.11, 4.62)**	**5.47 (5.02, 5.96)**	**3.86 (2.97, 5.02)**	
**Intracerebral hemorrhage**				
No. of cases	2,075	1,838	117	
<22	reference	reference	reference	
22-24	**1.64 (1.46, 1.84)**	**1.43 (1.27, 1.61)**	1.20 (0.65, 2.22)	**0.029**
≥25	**3.77 (3.26, 4.36)**	**2.97 (2.53, 3.48)**	**2.91 (1.46, 5.80)**	
**Subarachnoid hemorrhage**				
No. of cases	227	202	25	
<22	reference	reference	reference	
22-24	**1.75 (1.21, 2.53)**	**1.82 (1.28, 2.58)**	1.14 (0.26, 4.90)	0.380
≥25	**3.63 (2.33, 5.64)**	**2.28 (1.41, 3.69)**	1.13 (0.21, 5.97)	

Notes: aHR, adjusted hazard ratio. CI, confidence interval. AFLB, age at first live birth. Model was adjusted for age at baseline, residence, body mass index categories, waist circumference, smoking, passive smoking, drinking, physical activity, marital status, diabetes, hypertension, coronary heart disease, oral contraceptive pills usage, anticoagulation therapy, hypolipidemic therapy, menopausal status, parity, education, and annual household income. Significant interaction effects (P interaction <0.05) between AFLB and occupation were found for total stroke, ischemic stroke, and intracerebral hemorrhage.
